# Gene Manipulation Strategies to Identify Molecular Regulators of Axon Regeneration in the Central Nervous System

**DOI:** 10.3389/fncel.2017.00231

**Published:** 2017-08-07

**Authors:** Vinicius T. Ribas, Marcos R. Costa

**Affiliations:** ^1^Laboratory of Neurobiology, Department of Morphology, Institute of Biological Sciences, Federal University of Minas Gerais Belo Horizonte, Brazil; ^2^Brain Institute, Federal University of Rio Grande do Norte Natal, Brazil

**Keywords:** genetic manipulation, adeno-associated virus, transgenic animals, axon regeneration, central nervous system

## Abstract

Limited axon regeneration in the injured adult mammalian central nervous system (CNS) usually results in irreversible functional deficits. Both the presence of extrinsic inhibitory molecules at the injury site and the intrinsically low capacity of adult neurons to grow axons are responsible for the diminished capacity of regeneration in the adult CNS. Conversely, in the embryonic CNS, neurons show a high regenerative capacity, mostly due to the expression of genes that positively control axon growth and downregulation of genes that inhibit axon growth. A better understanding of the role of these key genes controlling pro-regenerative mechanisms is pivotal to develop strategies to promote robust axon regeneration following adult CNS injury. Genetic manipulation techniques have been widely used to investigate the role of specific genes or a combination of different genes in axon regrowth. This review summarizes a myriad of studies that used genetic manipulations to promote axon growth in the injured CNS. We also review the roles of some of these genes during CNS development and suggest possible approaches to identify new candidate genes. Finally, we critically address the main advantages and pitfalls of gene-manipulation techniques, and discuss new strategies to promote robust axon regeneration in the mature CNS.

## Introduction

Damage to the adult mammalian central nervous system (CNS), including acute brain or spinal cord injury, stroke, and neurodegenerative diseases, usually lead to permanent cognitive, sensory and/or motor disabilities. Axon regeneration failure is largely responsible for these long-term deficits and poor functional recovery. The promotion of robust axon regeneration in adult mammalian CNS holds great therapeutic potential for neurological disorders and is one of the major challenges in neuroscience. Moreover, the success of cell-based therapies to treat neurological disorders relies on the capacity of new neurons to grow processes and establish new synaptic contacts.

Numerous studies have focused on the characterization of the molecular mechanisms responsible for regenerative failure. In the beginning of the twentieth century seminal studies by Santiago Ramon y Cajal showed that axon fail to regenerate in the injured mature mammalian CNS (Ramon y Cajal, 1928), leading to the Ramon y Cajal's statement “once the development has ended, the founts of growth and regeneration of the axons and dendrites dried up irrevocably. In the adult centers the nerve paths are something fixed, ended and immutable. Everything may die, nothing may be regenerated. It is for science of the future to change, if possible, this harsh decree.” Many years later, Aguayo and his colleagues showed that after providing permissive substrates composed of peripheral nervous system (PNS) “bridges,” CNS axons are able to regenerate their axons (Richardson et al., 1980; Aguayo et al., 1981; David and Aguayo, 1981). These findings suggest that the CNS environment surrounding the injured adult neuron contains inhibitory factors that block regrowth.

Based on these initial reports, several research groups attempted to identify such factors and described a variety of extracellular inhibitory molecules expressed by the adult CNS oligodendrocytes and reactive astrocytes, including Nogo, myelin-associated glycoprotein (MAG), oligodendrocyte myelin glycoprotein (OMGP), chondroitin sulfate proteoglycans (CSPGs), semaphorins, and ephrins (Schwab and Bartholdi, 1996; Case and Tessier-Lavigne, 2005; Harel and Strittmatter, 2006; Yiu and He, 2006; Fitch and Silver, 2008; Schwab and Strittmatter, 2014; Silver et al., 2015). However, counteracting or removing extracellular inhibitory molecules results in limited and incomplete axon regeneration (Zheng et al., 2003; Liu et al., 2011; Mar et al., 2014; He and Jin, 2016).

Unlike adult CNS neurons, developing mammalian neurons can regenerate their axons after injury (Kalil and Reh, 1979), and grow new ones when transplanted into the injured adult CNS (Houlé and Reier, 1988), suggesting that mature mammalian CNS neurons have an intrinsically low capacity for axon growth compared to developing CNS neurons. Moreover, these findings indicate that neurons having a high capacity of axon growth are able to overcome the inhibitory environment of injured adult CNS. The capacity of axon regrowth declines greatly as neurons in the CNS mature. For instance, embryonic retinal ganglion cells have a great ability to regenerate their axons after lesion, which is dramatically reduced after birth (Bandtlow and Löschinger, 1997; Goldberg et al., 2002). Similar to developing CNS neurons, injured adult PNS neurons also have the capacity to grow their injured axons (Huebner and Strittmatter, 2009). These findings imply that the intrinsic capacity of axon outgrowth is a key feature regulating axon regeneration. This high intrinsic capacity of axon regrowth of developing CNS and adult PNS neurons is likely due to the expression of pro-regenerative genes, which allows the regeneration of damaged axons.

In the last decade, a considerable amount of research has been focused on the identification of genes regulating the intrinsic regenerative capacity of CNS neurons. Gene manipulation techniques have been widely used to modulate the expression of these genes in order to promote axon regrowth. Here, we review various studies that used genetic manipulation to foster axon regeneration after a variety of trauma to adult mammalian CNS (Table 1). We will first introduce a few commonly used gene manipulation strategies and the two most used experimental models to study axon regeneration in the adult CNS. Then, we will discuss the main findings involving the manipulation of specific genes and, based on their developmental roles, consider the possible pro-regenerative mechanisms that are triggered upon modulation of such genes.

**Table 1:** Summary of studies using genetic manipulation techniques to promote regeneration.

**Target**	**Modulation**	**Methods**	**Neuron type/model**	**Main effects**	**References**
PTEN	Deletion	AAV-Cre/“floxed” mice	RGC/optic nerve lesion	Enhanced axon regeneration	Park et al., 2008
	Deletion	AAV-Cre/“floxed” mice	CSN/spinal cord lesion	Enhanced axon regeneration and sprouting	Liu et al., 2010; Danilov and Steward, 2015; Du et al., 2015
	Knockdown	AAV-shRNA	CSN/spinal cord lesion	Enhanced axon regeneration	Zukor et al., 2013; Lewandowski and Steward, 2014
SOCS3	Inhibition (dominant negative)	Lentiviral vector	DRG neuron *in vitro*	Increased neurite growth	Miao et al., 2006
	Deletion	AAV-Cre/“floxed” mice	RGC/optic nerve lesion	Enhanced axon regeneration	Smith et al., 2009
	Overexpression	AAV	RGC/optic nerve lesion	Decreased axon regeneration	Hellström et al., 2011
STAT3	Overexpression	Lentiviral vector	DRG neuron *in vitro*	Increased neurite growth	Miao et al., 2006
	Overexpression	AAV	DRG neuron/spinal cord lesion	Enhanced axon regeneration and sprouting	Bareyre et al., 2011
	Overexpression	AAV	CSN/spinal cord lesion	Enhanced axonal sprouting	Lang et al., 2013
	Overexpression (hyperactive)	AAV	RGC/optic nerve lesion	Enhanced axon regeneration	Mehta et al., 2016
	Deletion	AAV-Cre/“floxed” mice	RGC/optic nerve lesion	Decreased axon regeneration	Sun et al., 2011
KLF4	Deletion	Thy1-Cre/“floxed” mice	RGC/optic nerve lesion	Enhanced axon regeneration	Moore et al., 2009
KLF7	Overexpression (hyperactive)	AAV	CSN/spinal cord lesion	Enhanced axon regeneration and sprouting	Blackmore et al., 2012
CREB	Constitutively active	Adenovirus	DRG neuron/spinal cord lesion	Enhanced axon regeneration	Gao et al., 2004
	Inhibition (dominant negative)	Retrovirus	Cortical neuron *in vitro*	Decreased neurite growth	Landeira et al., 2016
c-Jun	Deletion	Nestin-Cre/“floxed” mice	facial motoneurons/axotomy	Decreased axon regeneration	Raivich et al., 2004
	Knockdown	Electroporation of siRNA	DRG neuron/sciatic nerve lesion	Decreased axon regeneration	Saijilafu et al., 2011
	Overexpression	Lentiviral vector	DRG neuron *in vitro*	Increased neurite growth	Chandran et al., 2016
ATF3	Overexpression	Transgenic mice	DRG neuron/sciatic nerve lesion	Enhanced axon regeneration	Seijffers et al., 2007
SOX11	Overexpression	AAV	CSN/spinal cord lesion	Enhanced axon regeneration and sprouting	Wang et al., 2015
ASCL1	Overexpression	AAV	Brainstem neurons/spinal cord lesion	Enhanced axon regeneration	Williams et al., 2015
c-Myc	Overexpression	AAV and tamoxifen-inducible expression	RGC/optic nerve lesion	Enhanced axon regeneration	Belin et al., 2015
ROCK	Inhibition (dominant negative)	Lentiviral vector	RSN/spinal cord lesion	Enhanced axon regeneration	Wu et al., 2009
	Knockdown	AAV-shRNA	RSN/spinal cord lesion	Enhanced axonal sprouting	Challagundla et al., 2015
	Knockdown	AAV-shRNA	RGC/optic nerve lesion	Enhanced axon regeneration	Koch et al., 2014a
RhoA	Knockdown	AAV-shRNA	RGC/optic nerve lesion	Enhanced axon regeneration	Koch et al., 2014b
PTEN/SOCS3	Co-deletion	AAV-Cre/“floxed” mice	RGC/optic nerve lesion	Synergistic effect in increasing axon regeneration	Sun et al., 2011
	Co-deletion	AAV-Cre/“floxed” mice	CSN/spinal cord lesion	Synergistic effect in increasing axonal sprouting	Jin et al., 2015
SOCS3/KLF4	Co-deletion	AAV-Cre/“floxed” mice	RGC/optic nerve lesion	Synergistic effect in increasing axon regeneration	Qin et al., 2013
PTEN/SOCS3/c-Myc	Co-deletion of PTEN/SOCS3 and overexpression of c-Myc	AAV-Cre/“floxed” mice and AAV-c-Myc	RGC/optic nerve lesion	Synergistic effect in increasing axon regeneration	Belin et al., 2015
STAT3/ROCK	Overexpression of STAT3 and inhibition of ROCK	AAV-STAT3 and pharmacological inhibition	RGC/optic nerve lesion	Synergistic effect in increasing axon regeneration	Pernet et al., 2013
Rheb1/neural activity	Overexpression of Rheb1 and visual stimulation	AAV-Rheb1 and high-contrast images	RGC/optic nerve lesion	Synergistic effect in increasing axon regeneration	Lim et al., 2016

*RGC, retinal ganglion cell; CSN, corticospinal neuron; DRG, dorsal root ganglia*.

## Gene manipulation techniques in the CNS

Genetic manipulation approaches are particularly advantageous for studying mechanisms controlling axon regeneration, because it is possible to target and manipulate specific intracellular signaling molecules in particular neuronal types. Manipulations using viral vector-mediated gene transfer have been widely used to investigate the role of specific genes during different events that occur after CNS injuries (Table 1). In addition, the delivery of therapeutic genes to the CNS using viral vectors is also considered a valuable tool to potentially treat a number of incurable neurological disorders (Kaplitt et al., 1994; Burger et al., 2005; Cideciyan et al., 2009). Viral vectors have many advantages compared to other techniques to manipulate intracellular molecules, including delivery of recombinant proteins, naked DNA, or pharmacological substances, because it can provide long-term gene expression and targeting of specific neurons (Kaplitt et al., 1994; Klein et al., 1998; Kügler et al., 2003). The most used viral vectors for gene transfer in CNS neurons are lentiviral and recombinant adeno-associated viral (AAV) vectors. Lentiviral vectors are enveloped retroviruses containing a positive, single-stranded RNA genome, capable of infecting both dividing and non-dividing cells and provide long-term gene expression via integration into the host cells' genome (Ponder, 2000; Segura et al., 2013). However, one important disadvantage of lentiviral vectors is the potential of integration into active gene loci, which can result in insertional mutagenesis and the formation of tumors (Li et al., 2002; Schröder et al., 2002; Hacein-Bey-Abina et al., 2003; Themis et al., 2005). In addition, the production of lentiviral vectors is much more complex and their large diameter (~80–100 nm) can influence their distribution into the host tissue (Vogt and Simon, 1999; Segura et al., 2013).

In the last decades, recombinant AAV vectors have emerged as a particularly promising gene delivery method into the CNS. The AAV is a small (~25 nm), non-enveloped virus containing a linear single-stranded DNA genome (~4.7 kb; Berns and Giraud, 1996). AAV vectors provide long-term expression, are not associated with human diseases, have a low risk of insertional mutagenesis, low immunogenicity and have the ability to efficiently transduce a variety of neurons (Kaplitt et al., 1994; Papale et al., 2009; McCown, 2011). There are 11 naturally occurring AAV serotypes and even more variants (Wu et al., 2006), which express different capsid proteins that interact with a variety of receptors on target cells, resulting in distinct cellular tropism and different kinetics of transgene expression (Rabinowitz et al., 2002; Burger et al., 2005; Cearley and Wolfe, 2006; Vandenberghe et al., 2009). This, together with the route administration, allows the user to select the most appropriate AAV serotype to transduce the neuronal cell type of interest (Auricchio et al., 2001; Allocca et al., 2007; Lebherz et al., 2008; Hutson et al., 2012). The main drawback of AAV vectors, however, is the relatively small size of the internal payload, with a maximum capacity of ~4.7 kb (Gray et al., 2011; Hastie and Samulski, 2015). Nevertheless, recombinant AAV vectors have one of the best characterized safety profile, and have emerged as an appropriate delivery method for gene therapy into the CNS leading to an increasing number of clinical trials, including recent successes for the retinal degenerative disorder Leber's congenital amaurosis type 2 (Mandel and Burger, 2004; Cideciyan et al., 2009; Vandenberghe et al., 2009; Lim et al., 2010; Simonelli et al., 2010; Mingozzi and High, 2011; Testa et al., 2013). Therefore, AAV vectors are the method of choice to evaluate the role of specific genes and their products in axon regeneration.

Another common strategy used to manipulate gene expression in animal (specially mice) models of CNS injuries is the so-called Cre/lox system (Sauer, 1998). This is a site-specific recombination system based on the activity of the enzyme Cre recombinase (CRE), which recognizes specific 34 bp DNA sequences called loxP sites. LoxP sites are added to a transgene so that they flank a sequence of DNA, referred to as a “floxed” sequence. The enzyme CRE recognizes these loxP sites and either excise or invert DNA sequences depending on the loxP sites orientation. Transgenic mice can be engineered to express a transgene under the control of CRE. For that, a floxed stop codon is placed directly upstream of the transgene, preventing its expression. Following CRE expression, the stop codon is removed and the transgene can be expressed. Transgenic mice can also be engineered to insert loxP sites into specific endogenous genes. In these animals, CRE activity leads to the excision/inversion of DNA sequences with the consequent loss of the targeted genes. Thus, the Cre/lox system can be used to either induce or inhibit gene expression.

Tissue specific recombination can be achieved through the use of transgenic lines in which CRE expression is regulated by cell-type specific promoters. Moreover, transgenic animals carrying the gene for an enzyme CRE fused to the truncated estrogen receptor (ERT) allows the temporal control of CRE-mediated DNA recombination, since the nuclear transport of the fused CreERT requires the treatment with the estrogen analog Tamoxifen. For example, crossing transgenic mice expressing CreERT in neurons (Young et al., 2008) to transgenic mice carrying a “floxed” sequence in the *pten* gene (Park et al., 2008) would allow the temporal deletion of this gene at different time-points after axonal injury. This strategy of gene deletion is called “conditional knockout.”

Alternatively, CRE expression can be temporally and spatially controlled through the use of viral vectors carrying plasmids encoding for CRE under the control of cell-specific promoters. Most of the studies using AAV vectors to express CRE, in models of CNS injury, used ubiquitous promoters, such as cytomegalovirus (CMV; Park et al., 2008; Du et al., 2015; Geoffroy et al., 2015) and the hybrid CMV/chicken ß-actin (CAG) promoters (Bei et al., 2016), or neuronal specific promoters such as human synapsin (hSyn) (Qin et al., 2013). In addition to CRE, expression of therapeutic genes delivered by viral vectors can also be regulated through the use of cell-specific promoters.

The use of ubiquitous promoters has important caveats due to transgene expression in undesired cell types, such as local inhibitory neurons and glial cells (Nathanson et al., 2009; Watakabe et al., 2015, 2017). Moreover, it has been shown that high levels of transgene expression, driven by CMV promoter, may be toxic to neurons (Watakabe et al., 2015). The use of neuronal-specific promoters, such as hSyn, mouse-calcium/calmodulin-dependent protein kinase II (CAMKII), platelet-derived growth factor-ß chain (PDGF-ß), neuron-specific enolase (NSE), among others, can avoid expression of transgene in non-neuronal cells and also the toxic effect produced by high levels of transgene expression (Klein et al., 1998; Paterna et al., 2000; Kügler et al., 2003; Nathanson et al., 2009; McLean et al., 2014; Watakabe et al., 2015). Nonetheless, hSyn promoter controlled gene expression in the CNS leads to transgene expression in both inhibitory and excitatory neurons, whereas CAMKII promoter tend to be more specific to excitatory neurons (Watakabe et al., 2015). Similar to neurons, glial cells can also be targeted by using promoters of glia-specific genes, such as myelin basic protein (*Mbp*) for oligodendrocytes or glial fibrillary acidic protein (*Gfap*) for astrocytes (Lawlor et al., 2009; von Jonquieres et al., 2013). Thus, through the choice of different promoters, transgene expression can be selectively induced in distinct cell types in the CNS, which allows the evaluation of the contribution of different genes in specific cell types to axon regeneration.

## Experimental CNS injury models

Many different injury models are used to study axon regeneration in the CNS. Nonetheless, optic nerve lesion and spinal cord injury are the most widely used experimental strategies and will be the focus of this review. The optic nerve is part of the CNS and contains the axons of retinal ganglion cells (RGCs), the sole output of the retina. The axons of the optic nerve project to different brain areas, including the dorsal lateral geniculate nucleus, superior colliculus, and suprachiasmatic nucleus, among others. Like other areas of the CNS, optic nerve axons cannot regenerate after injury. Therefore, conditions such as glaucoma, that involves degeneration of these axons, leads to irreversible vision impairment. The optic nerve lesion model has many advantages, such as the simple anatomy (i.e., all lesioned axons belong to only one cell type), easy accessibility and the functional relevance. Moreover, gene manipulation in retinal cells, including RGCs, using viral vectors, has numerous benefits. First, the anatomical organization of the retina in which the cells are organized in different layers facilitate the transduction of specific neuronal cell types (i.e., for RGC transduction intravitreal injection is performed, while for photoreceptor transduction sub-retinal injection is more appropriated). Second, the structure and accessibility of the retina allow easy access to local administration of viral vectors. Finally, the eye is a compartmentalized structure and, due to the presence of the blood–retinal barrier, prevents the unintentional systemic spread of vectors, thus limiting immune responses toward the transgene and the vector proteins (Bennett, 2003; Streilein, 2003). Due to these advantages, AAV-mediated gene therapy is a common strategy employed in animal models to treat retinal diseases (Liang et al., 2001; Dejneka et al., 2004; Acland et al., 2005; Mingozzi and High, 2011), indicating that gene manipulation using these vectors to promote axon regeneration could be a valuable strategy for clinical applications. Therefore, the optic nerve lesion model has been widely used to investigate the role of specific genes in axon regeneration in the CNS.

Spinal cord injury is another widely model to study axon regeneration in the CNS. Traumatic spinal cord injuries in humans can often result in permanent sensory and/or motor deficits (Yip and Malaspina, 2012), that cannot be sufficiently repaired by any existing therapy. One of the major challenges to achieve functional recovery after such injuries is to promote robust axonal regeneration. Usually, in animal models of spinal cord injury, the main focus is pyramidal corticospinal neurons (CSNs). CSNs are localized in the layer V of the primary motor cortex and send their axons directly to the spinal cord, where the majority of the axons form the dorsal corticospinal tract (CST), which in rodents runs in the ventral portion of the dorsal funiculus. The CST is an important descending motor pathway that contains the axons of neurons controlling locomotion, posture, and voluntary skilled movements, especially in the distal part of the limbs (Steward et al., 2004; Lemon and Griffiths, 2005). Damage to CST axons usually occurs after spinal cord lesion and lead to permanent motor deficits. Notably, CSNs have a particularly low capacity for axon regrowth, with minimal regeneration even after permissive tissue grafts or neutralization of extracellular inhibitory molecules (Richardson et al., 1980; Hollis et al., 2009; Lee et al., 2010). Thus, the promotion of CST regeneration remains a fundamental step to restore motor function after this type of injury.

Gene manipulation in CSNs is a common strategy to evaluate the role of genes in CST axon regeneration. To this end, most studies use AAV vectors, which are injected into the sensorimotor cortex to transduce CSNs and modulate gene expression in this neuronal type. In addition to CST axons, the spinal cord injury model is also used to evaluate axon regeneration of other axonal tracts, such as rubrospinal tract in the dorsal part of the lateral column, which is also involved in motor control, and ascending sensory axons in the dorsal column that mediate sensorial stimuli. Although, gene manipulation of these other pathways could be used to study axon regeneration in spinal cord injury models, most of the studies focused on the CST. One important disadvantage of spinal cord injury models is that usually the animals require extensive post-operative care, including deliver of fluids, pain medications, and bladder emptying to avoid urinary tract infections.

## PTEN/mTOR

**Figure 1:**
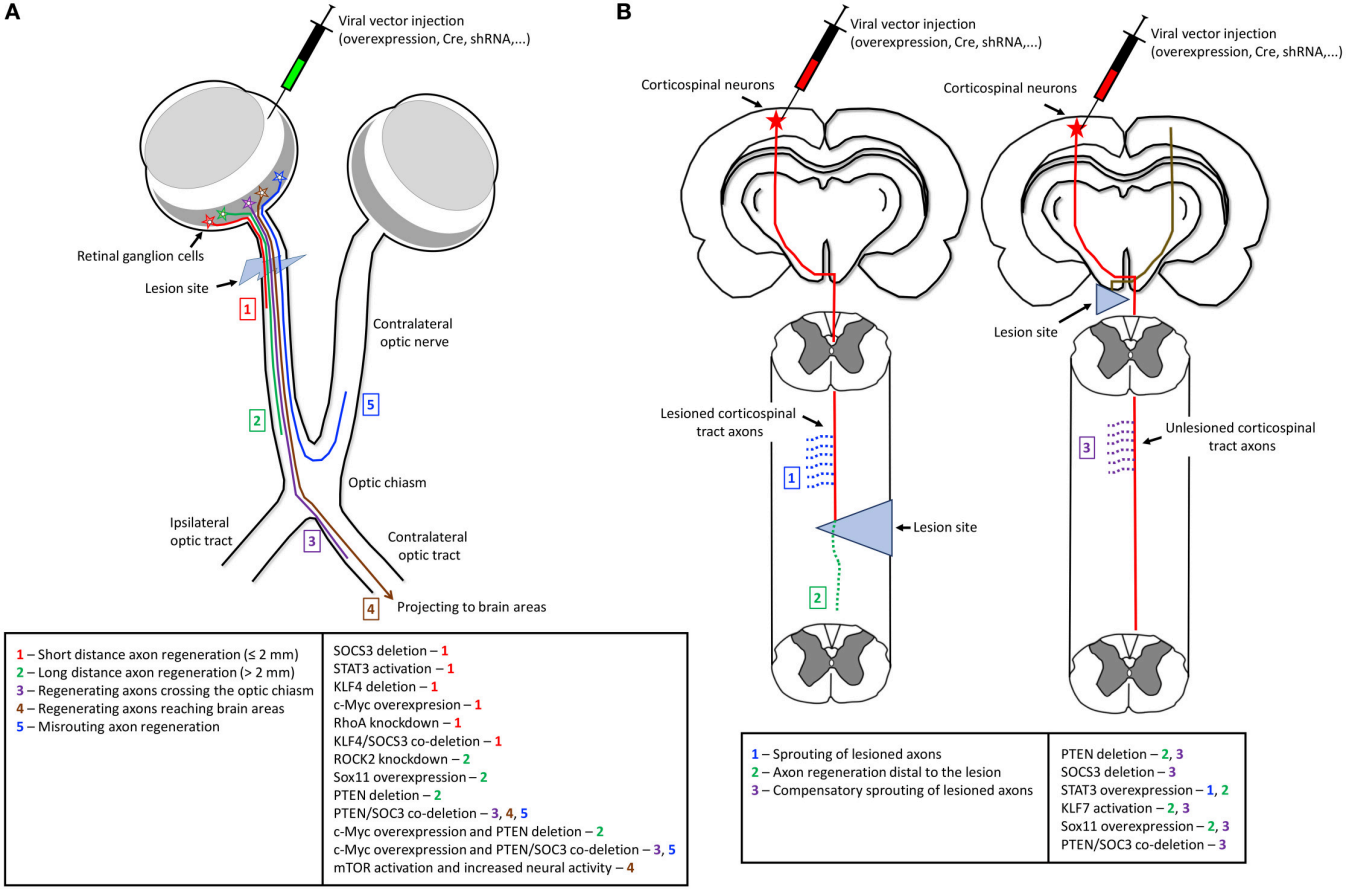
The effects of gene manipulation on axon regeneration in optic nerve and spinal cord lesion models of CNS injury. The main technique to manipulate gene expression is by direct injections of viral vectors to overexpress specific genes, deliver Cre recombinase for gene deletion, or deliver shRNA against endogenous targets for gene knockdown. **(A)** In the optic nerve lesion model genetic manipulation is usually by intravitreal injections. The effects of different genes manipulation (right box) range from short distance axon regeneration up to complete axon regrowth to target areas (numbers—left box). **(B)** In spinal cord injury model, genetic manipulation is usually by injections into the sensorimotor cortex to transduce corticospinal neurons. The effects of manipulation of specific genes (right box) varies from sprouting of lesioned axons (1), axon regeneration distal to the lesion (2), and compensatory sprouting of unlesioned axons (3) (left box). The left scheme shows gene manipulation in lesioned neurons of a spinal cord injury model. The right scheme depicts a unilateral pyramidotomy and gene manipulation performed in unlesioned neurons.

To date, an increasing number of studies have targeted neuron-intrinsic molecules and signaling pathways in order to increase the capacity of injured adult mammalian CNS neurons to regenerate their axons (Table 1). PTEN/mTOR pathway manipulation has resulted in one of the most robust effect on axon regeneration (Figure 1). PTEN (phosphatase and tensin homolog) is a phosphatase that inhibits protein kinase B (PKB, also termed Akt) activity through conversion of phosphatidylinositol (3,4,5) trisphosphate (PIP3) to phosphatidylinositol (4,5) bisphosphate (PIP2) (Guertin and Sabatini, 2007; Carnero, 2010). On the contrary, phosphoinositide 3-kinase (PI3-K) converts PIP2 to PIP3 and activates Akt, which activates the mechanistic target of rapamycin (mTOR) (Luo et al., 2003). Thus, inactivation of PTEN induces Akt activity culminating in the activation of mTOR, which is a central regulator of cellular protein synthesis, autophagy, growth and survival (Chong et al., 2012; Saxton and Sabatini, 2017). Activation of mTOR promotes protein synthesis mainly by phosphorylating the kinase S6K and the translation regulator 4E-BP1 (Ma and Blenis, 2009). In addition to promoting protein synthesis, mTOR also inhibits catabolism by blocking autophagy through the phosphorylation of the ULK1–Atg13–FIP200 complex (Jung et al., 2009). Therefore, mTOR is an important regulator of anabolism and catabolism mechanisms that could play an important role on axon regeneration.

In the developing brain, PTEN/mTOR pathway play an important role on the control of stem cell/progenitor proliferation (Groszer et al., 2001; Lehtinen et al., 2011), neuronal differentiation, migration, and process growth (Kwon et al., 2006; Hsia et al., 2014; Morgan-Smith et al., 2014). More importantly to our discussion, downregulation of PTEN in developing neurons leads to increased axonal branching and growth (Drinjakovic et al., 2010). Similarly, PTEN inhibition enhances neurite outgrowth in neurons derived from human embryonic stem cells (Wyatt et al., 2014). These observations suggest that regulation of PTEN/mTOR signaling is key to control axonal growth during development and, therefore, could be a potential target to foster regeneration in the injured adult CNS.

According to this notion, a leading study showing the effects of PTEN deletion on axon regeneration was published in 2008 (Park et al., 2008). In this study they used conditional knockout mice in which the gene encoding PTEN protein was flanked by loxP sites. They deleted PTEN in RGCs by intravitreal injection of AAV2 particles expressing Cre recombinase (AAV-Cre) in adult mice. PTEN deletion resulted in robust axon regeneration after optic nerve lesion, with a large number of regenerating axons elongating up to 4 mm distal to the lesion site. In addition to axon regeneration, PTEN deletion also increased RGC survival after optic nerve lesion, with a protection rate of about two-fold compared to control. Both, axon regeneration and RGC survival were reduced by application of rapamycin, an inhibitor of mTOR, suggesting that the observed effects induced by PTEN deletion are dependent on mTOR pathway (Park et al., 2008).

After these pioneer findings, several studies examined the effects of PTEN deletion in different models of CNS injury. In a follow up study from the same research team, the effect of PTEN deletion on axon regeneration was also investigated in models of spinal cord injury. Using the same approach, PTEN was deleted by injecting AAV-Cre into the sensorimotor cortex of neonatal mice in order to evaluate CSNs axon regeneration. As observed in RGCs, PTEN deletion induced robust axon regeneration after spinal cord injury, both, by enhanced compensatory sprouting of uninjured CSN axons and successful regeneration of injured CSN axons distal to the lesion site (Liu et al., 2010). On both studies, the authors observed a reduction of mTOR activity in adult CNS neurons as compared to the developing CNS, and axonal lesion further reduced this activity (Park et al., 2008; Liu et al., 2010). This suggests that mTOR is a possible intrinsic regulator of axon growth which could be developmentally downregulated. Regarding the molecular mechanisms downstream of mTOR which could regulate axon regeneration, it is believed that enhancement of protein synthesis induced by mTOR activity could provide building blocks for axonal regrowth and would be the main reason for the increased axon regeneration. However, whether other downstream targets of mTOR, including autophagy, are also involved in the regulation of axon regeneration following PTEN deletion remains to be determined.

In addition to these two publications, other studies also showed that PTEN is in fact a valuable target to promote axon regeneration. Two independent studies using AAVs to deliver short hairpin RNAs (shRNA) against PTEN to CSNs, have also shown that PTEN downregulation enhances CST axon regeneration and improves the recovery of skilled motor functions after spinal cord injury in rodents (Zukor et al., 2013; Lewandowski and Steward, 2014). Moreover, conditional deletion of PTEN in CSNs enhances regrowth of CST axons and motor function recovery even after the spinal cord injury (Danilov and Steward, 2015). Notably, PTEN deletion one year after spinal cord injury still triggers robust CST regeneration (Du et al., 2015).

Although, these studies do not describe any major detectable pathology, long-term effect of PTEN interference is a potential concern, because PTEN is a tumor suppressor, whose manipulation could lead to undesirable side effects. Moreover, PTEN deletion in hippocampal dentate granule cells leads to mTOR hyper activation and promotes the rapid onset of spontaneous seizures (LaSarge et al., 2015). In a recent study, Gutilla and colleagues evaluate the long-term effect of neonatal PTEN deletion in mice. Albeit the authors found no evidence of tumor formation or other major neuropathology, cortical thickness was significantly increased and cortical lamination was disrupted in the area of PTEN deletion (Gutilla et al., 2016). However, the authors did not investigate the long-term effect of PTEN deletion in adult animals. Therefore, PTEN interference could be a potential pro-regenerative strategy for treating CNS injuries, but systematic studies are necessary to prove the long-term safety of PTEN interference.

## JAK/STAT3/SOCS3

Several studies using gene manipulation techniques have indicated that the JAK/STAT3/SOCS3 pathway is also an important modulator of axon regeneration (Table 1; Figure 1; Liu et al., 2015). In this pathway, cytokines, including interleukin-6 (IL6) and ciliary neurotrophic factor (CNTF), bind to receptors and activate the Janus kinase (JAK)/signal transducer and activator of transcription (STAT) pathway (Krebs and Hilton, 2001), a critical intracellular cascade for the transduction of extracellular signals to the nucleus. Upon binding of the ligand to its receptor, JAK phosphorylates STATs, including STAT3, which dimerizes and translocate to the nucleus, where it interacts with various regulatory elements that induce target gene expression (Darnell et al., 1994). The above signaling cascades transcriptionally regulates a family of proteins called Suppressor of cytokine signaling (SOCS), including SOCS3, which act as a negative feedback signal by inhibiting JAK and STAT activation and phosphorylation, limiting the response to cytokine and growth factors signaling (Krebs and Hilton, 2001).

One of the first evidence showing that the JAK/STAT3/SOCS3 pathway regulates axon regeneration was observed in rat primary sensory neurons *in vitro* (Miao et al., 2006). In this study, they showed that overexpression and activation of STAT3, by lentiviral transduction, stimulates neurite growth of cultured sensory neurons. Overexpression of SOCS3 blocks nuclear translocation of STAT3 and neurite outgrowth, and inhibition of endogenous SOCS3 through overexpression of a dominant negative mutant SOCS3 stimulates neurite outgrowth (Miao et al., 2006). Thereafter, Smith and colleagues showed, using the *in vivo* AAV-mediated conditional knockout approach discussed above, that deletion of SOCS3 in adult RGCs is able to promote extensive axon regrowth after optic nerve lesion. This effect was abolished by co-deletion of gp130, a shared receptor component required for this signaling, indicating that the regenerating effect of SOCS3 deletion is dependent on gp130. Moreover, injection of CNTF in SOCS3 deleted mice further increase the extent of axon regeneration (Smith et al., 2009). Consistent with these findings, overexpression of SOCS3 in RGCs, by using AAV vectors, inhibited regeneration of optic nerve axons induced by CNTF injection or peripheral nerve grafting (Hellström et al., 2011). Altogether, these studies point to SOCS3 as a pivotal negative regulator of JAK/STAT3/SOCS3 pathway in regulating axon regeneration.

Several studies suggest that STAT3 is the primary effector of axon regeneration induced by this pathway. In a study combining *in vivo* time-lapse fluorescence microscopy with genetic manipulations in mice, the authors showed that selective deletion of STAT3 in dorsal root ganglion (DRG) neurons impairs regeneration of peripheral DRG branches after nerve cut. In addition, overexpression of STAT3 increases outgrowth and collateral sprouting of central DRG branches after a dorsal column lesion (Bareyre et al., 2011). Thereafter, another study from the same group demonstrated that overexpression of STAT3 in CSNs enhances remodeling of lesioned CST axons and induces axonal sprouting from unlesioned CST axons, leading to functional recovery (Lang et al., 2013). More recently, Mehta and colleagues have shown that overexpression of STAT3 fused with a viral activation domain (VP16), which hyper activates the transcription activity of STAT3, in RGC results in regeneration of optic nerve axons after injury (Mehta et al., 2016). Finally, AAV-mediated conditional knockout study demonstrated that SOCS3 deletion leads to significant axon regeneration in the optic nerve, while double SOCS3/STAT3 deletion reverts the regenerative effects of SOCS3 deletion alone (Sun et al., 2011). These data indicate that STAT3 is the critical mediator of axon regeneration induced by SOCS3 deletion.

Interestingly, the JAK/STAT pathway also regulates the generation of astroglial cells in the developing CNS (Bonni et al., 1997; Miller and Gauthier, 2007). Timewise, axon growth and astrogliogenesis partly overlap in the developing CNS, suggesting that these two processes are intermingled and could be manipulated to further stimulate axon growth in the injured adult CNS. According to this notion, RNA-interference (RNAi) of GFAP and Vimentin expression leads to suppression of astroglial reactivity and scarring, and increase survival and neurite growth of cortical neurons in culture (Desclaux et al., 2009).

The JAK/STAT3/SOCS3 pathway, thus, appears to play a pivotal role in controlling axon growth, and represents a promising target for therapeutic intervention. Modulating this pathway after CNS injury could help axon regeneration and ultimately functional recovery. Similar to PTEN, however, SOCS3 acts a tumor suppressor, an effect likely mediated via the JAK/STAT pathway (He et al., 2003; Tokita et al., 2007). Therefore, additional studies are necessary to confirm that manipulation of this pathway can promote long-term regenerative benefits without leading to undesired side-effects in the injured CNS.

## KLF family of transcription factors

The members of the Krüppel-like factors (KLF) family of transcription factors have also been implicated in the control of axon regeneration (Table 1; Figure 1). Members of this family are involved in the loss of axon growth capacity observed in RGCs around birth (Goldberg et al., 2002; Moore et al., 2009). Indeed, KLF4 expression is upregulated in RGC after birth, and overexpression of this transcription factor in embryonic RGCs induces a potent reduction in neurite outgrowth *in vitro*, suggesting that this protein inhibits axon elongation in these cells. Accordingly, KLF4 knockout increases the number and length of regenerating RGC axons after optic nerve injury (Moore et al., 2009). In addition to KLF4, overexpression of other members of the KLF family, including KLF15, KLF9, KLF14, KLF13, KLF5, KLF12, and KLF1, also decreases neurite growth in cortical neurons *in vitro*. Similar to KLF4, KLF9 is clearly upregulated postnatally in RGCs (Moore et al., 2009). Moreover, overexpression of another member, KLF16, inhibits RGC neurite outgrowth *in vitro* and enhances RGC growth cone collapse in response to exogenous ephrinA5 ligands (Wang et al., 2016). In contrast, overexpression of KLF6 and KLF7 increases neurite growth in cultured postnatal RGCs (Moore et al., 2009). Similarly, overexpression of a chimeric KLF7 with the VP16 transactivation domain in CSNs, promotes both sprouting and regenerative axon growth in the CST of adult mice (Blackmore et al., 2012). These studies point to a complex role for the KLF family of transcription factors as regulators of axon regeneration in the CNS, by both promoting and inhibiting axon growth. Thus, manipulation of different members of this family can be potentially used to promote axon regrowth after CNS injury. Although, it is clear that this family of transcription factors is involved in the control of axon regeneration, the effects of KLF manipulations are relatively modest. In addition, little is known about protein-protein interactions of the members of this family that are relevant to axon elongation in the CNS. Understanding the crosstalk between these transcription factors may yield better strategies for promoting robust CNS axon regeneration.

## Other transcription factors (CREB/c-Jun/ATF3/SOX11/ASCL1/c-Myc)

The transcription factors cAMP Responsive Element Binding Protein (CREB), Jun Proto-Oncogene (c-Jun), Activating Transcription Factor 3 (ATF3), SRY (Sex Determining Region Y)-Box 11 (SOX11), Achaete-Scute Family BHLH Transcription Factor 1 (ASCL1), and the Proto-oncogene c-Myc, can also play a role on axon regeneration (Table 1; Figure 1).

The transcription factor CREB belongs to the family of leucine zipper transcription factors, which binds to the cAMP response element (CRE), a conserved DNA sequence in promoter elements that are activated by cAMP (Montminy and Bilezikjian, 1987). Overexpression of a dominant negative form of CREB in cerebellar and DRG neurons, blocks neurite growth induced by cAMP (Gao et al., 2004). Conversely, overexpression of a constitutively active form of CREB in DRG neurons, promotes regeneration of central DRG branches after a dorsal column lesion (Gao et al., 2004). Moreover, recent work from our laboratory has shown that CREB-mediated signaling is involved in neurite elongation of immature cerebral cortex neurons (Landeira et al., 2016), suggesting that this transcription factor represents a potential target to promote corticospinal axon regeneration.

The role of c-Jun transcription factor in axon regeneration has also been examined. Conditional knockout of c-Jun in facial motor neurons decreases axon regeneration of facial nerves after axotomy (Raivich et al., 2004). Moreover, knockdown of c-Jun in DRG neurons, using *in vivo* electroporation of siRNA, significantly impairs axon regeneration of peripheral branches after sciatic nerve lesion (Saijilafu et al., 2011), while overexpression of c-Jun in these cells is able to promote neurite outgrowth *in vitro* (Chandran et al., 2016). Although, c-Jun has a positive effect on axon regeneration, it has been shown that c-Jun expression in required for cell death of facial motoneurons after axotomy as well as in RGC after optic nerve lesion (Yoshida et al., 2002; Raivich et al., 2004; Lingor et al., 2005). Thus, these studies demonstrate that c-Jun, an immediate early gene, has a dual role in neurons, involved in the induction of axon regeneration and neuronal cell death. While deletion of c-Jun impaired axonal regrowth in the CNS and PNS, it remains to be tested whether forced expression of c-Jun improves axon regeneration in the CNS *in vivo*. In addition, the role of c-Jun on neuronal cell death precludes the use of c-Jun for clinical applications.

The transcription factor ATF3, which can form heterodimer with c-Jun, is also able to increase axonal regrowth in the PNS. In transgenic mice that constitutively express ATF3, there is an increase in axonal regeneration of DRG peripheral branches after injury (Seijffers et al., 2007). However, constitutively expression of ATF3 does not overcome myelin inhibition on neurite outgrowth of DRG in culture or enhance central axon regeneration in the spinal cord after dorsal column injury *in vivo* (Seijffers et al., 2007). These findings demonstrate that genes involved in axonal regrowth in the PNS are not necessary important for axon regeneration in the CNS. Nevertheless, it remains to be tested whether manipulations of c-Jun and ATF3 are effective in promoting axon growth in different CNS neuronal types after injury. Moreover, whether there is a crosstalk between c-Jun, ATF-3 and others transcription factors like ATF-2, which is relevant to axon regeneration remains to be determined.

SOX11 is an example of transcription factor required for PNS axon growth whose forced expression promotes regeneration in the CNS. SOX11 is highly expressed in the embryonic CNS and PNS, but it is downregulated at later developmental stages (Hargrave et al., 1997; Tanabe et al., 2003; Dy et al., 2008; Penzo-Méndez, 2010). However, its expression in rapidly upregulated in DRG neurons following peripheral axotomy, and its deletion decreases PNS neurite growth (Tanabe et al., 2003; Lin et al., 2011). Recently, Wang and colleagues showed that overexpression of SOX11 in CSNs is sufficient to promote CST compensatory sprouting and axon regeneration after spinal cord injury. The effect of SOX11 forced expression on axon regeneration was observed, although at lesser extent, even when delivered 2 months after injury, indicating that SOX11 is able to promote axon regrowth in both acute and chronic injury paradigms. Despite of that, the normal recovery of forelimb dexterity that occurs after cervical spinal cord injury was impaired in SOX11-treated animals on both injury paradigms (Wang et al., 2015). These data suggest that regenerating axons induced by SOX11 overexpression may not establish functional synapses, or they form synapses with wrong targets, which could prevent functional recovery. Considering the roles of SOX11 in the developing cerebral cortex as an upstream regulator of FEZF2, which is required for the specification of corticospinal neuron identity and connectivity (Shim et al., 2012; Muralidharan et al., 2017), and as a negative controller of dendritic morphogenesis (Hoshiba et al., 2016), it is possible that sustained expression of SOX11 in adult neurons leads to conflicting effects on axonal regeneration. According to this notion, overexpression of SOX11 in RGCs promotes robust axon regeneration in a subset of RGCs after optic nerve lesion, but also induces cell death in another subtype of RGC (Norsworthy et al., 2017). Future studies should help elucidating whether different levels of SOX11 expression are responsible for those conflicting effects in RGCs. Similarly, it remains to be tested whether a time-controlled expression of SOX11 could lead to robust axon regeneration while avoiding undesired effects.

ASCL1 is a proneural basic helix-loop-helix (bHLH) transcriptional factor involved in cell fate determination and differentiation (Vasconcelos and Castro, 2014). It has been recently shown that ASCL1 is required to axon regeneration of RGCs after optic nerve crush in adult zebrafish. Furthermore, overexpression of ASCL1 in brainstem neurons of rats promotes noradrenergic axon regeneration after spinal cord injury and improves hindlimb movement recovery (Williams et al., 2015). These findings suggest that overexpression of proneural genes in adult neurons after injury could be an interesting strategy to promote axon regeneration in the mammalian CNS. According to this notion, ASCL1 expression upregulates the expression of several genes involved in axonogenesis and axon guidance during neuronal differentiation (Castro et al., 2011; Raposo et al., 2015). Moreover, ASCL1 induces the expression of other transcription factors involved in axon regeneration, such as SOX11 (Masserdotti et al., 2015). Last but not least, binding of ASCL1 to DNA increases chromatin accessibility at regulatory regions of its target genes (Raposo et al., 2015), what could be an interesting mechanism to overcome epigenetic barriers to the expression of pro-regenerative genes after CNS injury.

A recent elegant study points to c-Myc as a pivotal transcription factor controlling axon regeneration and survival of RGC after optic nerve injury. The transcription factor c-Myc is a oncogene known to control cell cycle progression, proliferation, growth, adhesion, differentiation, apoptosis, and metabolism (Meyer and Penn, 2008). In adult mice, c-Myc is highly expressed in RGC and it is downregulated after optic nerve crush (Belin et al., 2015). Overexpression of c-Myc promotes RGC survival and axon regeneration after optic nerve crush. As an additional highlight of this study, the authors showed, using a tamoxifen-inducible c-Myc transgenic mouse line, that transient overexpression of c-Myc prior to optic nerve injury promotes both robust neuronal survival and axon regeneration of RGCs. Moreover, c-Myc expression even after injury significantly increases RGC survival and axon regeneration, suggesting that c-Myc can rescue these injured neurons from apoptotic death and promote their axon regeneration (Belin et al., 2015). Although, the authors showed very robust effects of neuronal survival and axon regeneration, additional long-term studies are necessary in order to translate this strategy to the clinics because c-Myc is a well know transcription factor involved in tumorigenesis.

## Rho/ROCK

Although, the main focus of this review is not extracellular inhibitory molecules that block axon regeneration, here we will briefly discuss some studies that manipulate the Rho/ROCK pathway, which is an important intracellular signaling pathway activated by different extracellular inhibitory factors (Shamah et al., 2001; Dontchev and Letourneau, 2003; Lin et al., 2007). Many of these inhibitory components bind to receptors in the axonal membrane and lead to activation of Ras homologous member A (RhoA) resulting in growth cone collapse and axonal retraction (Jalink et al., 1994; Wahl et al., 2000; Gu et al., 2013). The Rho-associated coiled-coil-containing protein kinase (ROCK) has been identified as a main downstream target of RhoA (Ishizaki et al., 1997), which is responsible for propagating this signal to the cytoskeleton, modulating neurite growth and axon regeneration (Moreau-Fauvarque et al., 2003; Mueller et al., 2005). Several studies have used genetic manipulation techniques to modulate this pathway to promote axonal regrowth. Expression of a dominant negative mutant of ROCK promotes neurite outgrowth of DRG neurons cultured on myelin substrate. In addition, expression of this dominant negative mutant in rubrospinal neurons enhances axon regeneration of rubrospinal tract (RST) axons after cervical spinal cord injury. Importantly, this was accompanied by recovery of forelimb and hindlimb functions (Wu et al., 2009). Focusing on the same neuronal type, we have shown that post-injury knockdown of ROCK2, using a shRNA against it, promotes rubrospinal neuron survival and prevents atrophy after spinal cord hemisection (Challagundla et al., 2015). Moreover, knockdown of ROCK2 enhances RST axonal sprouting proximal to the lesion. However, in this study ROCK2 downregulation did not increase axon regeneration distal to the lesion and only promotes minimal recovery in hindlimb motor behavior (Challagundla et al., 2015). In the visual system, knockdown of ROCK2 enhances neurite outgrowth of RGCs cultured on inhibitory substrates. Furthermore, knockdown of ROCK2 induces substantial axonal regeneration, increases survival of RGCs and attenuates axonal degeneration of proximal axons after optic nerve injury assessed by *in vivo* live imaging (Koch et al., 2014a). In addition to ROCK, downregulation of RhoA has beneficial effects on neuronal survival and regeneration. Knockdown of RhoA promotes neurite outgrowth of RGC neurons cultured on inhibitory substrate as well as neurite regeneration of primary midbrain neurons after scratch lesion. Besides that, downregulation of RhoA significantly enhances axonal regeneration and survival of RGCs after optic nerve lesion (Koch et al., 2014b). Taken together, these findings imply that the Rho/ROCK pathway may be an interesting molecular target for the treatment of traumatic CNS injury.

Considering the broad roles of RhoA/ROCK pathway in the control of cell proliferation, specification, survival and migration (Cappello, 2013; Duquette and Lamarche-Vane, 2014), it is possible that direct downregulation of RhoA or ROCK may lead to undesired effects. However, new approaches to interfere with specific effects of that pathway on axonal growth could be designed in the future. According to this notion, it has been shown that the ubiquitin E3 ligase Smad Ubiquitin Regulatory Factor 1 (Smurf1) is phosphorylated at Threonin 306 by PKA after BDNF treatment of hippocampal and cerebral cortex neurons. This phosphorylated form of Smurf1 then reduces degradation of polarity protein Par6 and increases degradation of growth-inhibiting RhoA, leading to neuronal polarity and axon initiation (Cheng et al., 2011). These observations suggest that site-specific phosphorylation of ubiquitin E3 ligases could be a useful mechanism for establishing specific spatiotemporal patterns of RhoA/ROCK (and other proteins) expression that are required for axonal growth without interfering with other cell functions.

## Combinatorial strategies

**Figure 2:**
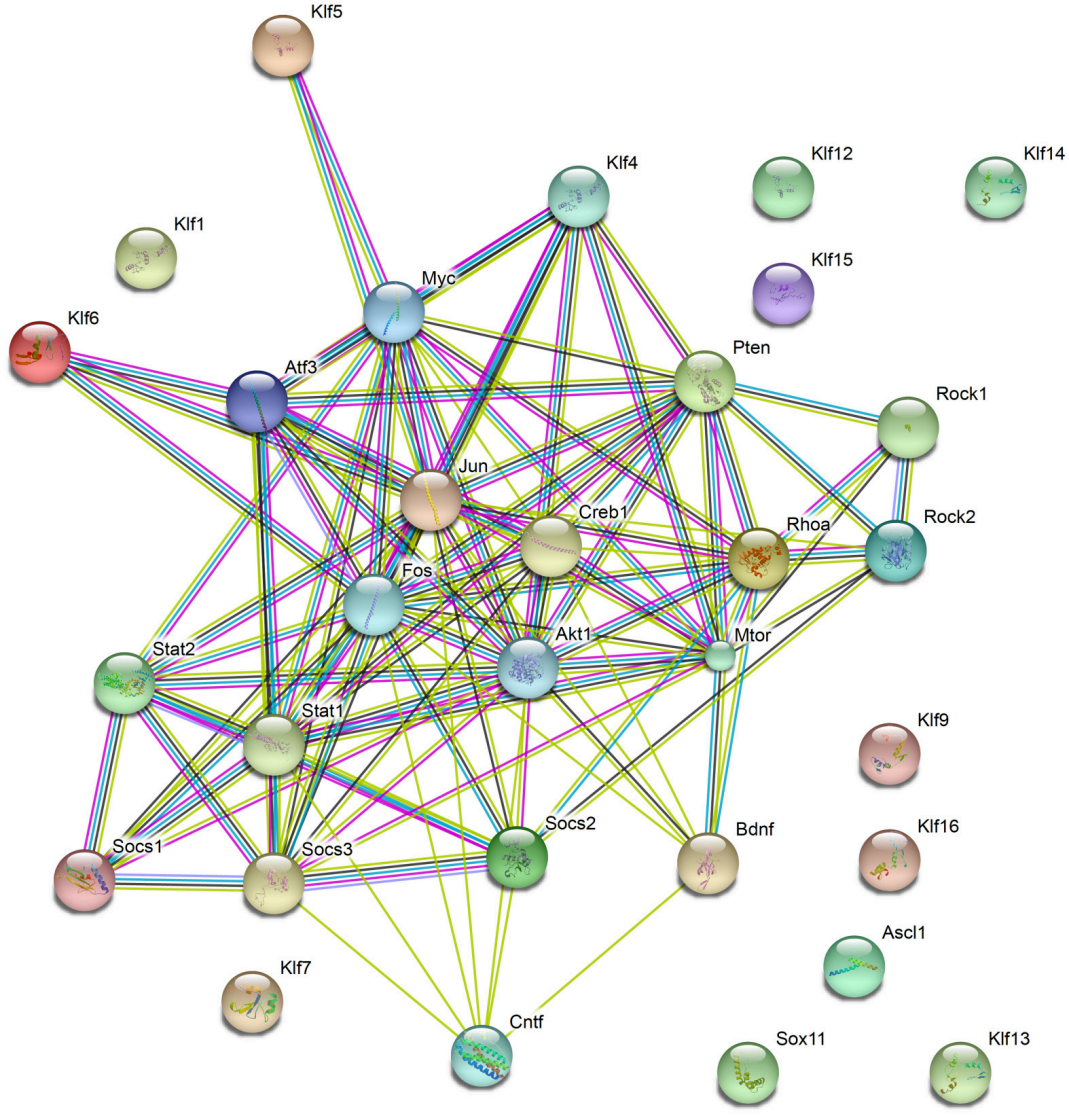
Functional protein networks involved in axon regeneration. Schematic representation of interactions among proteins manipulated to stimulate CNS axon regeneration and discussed in this work. Nodes show the proteins described throughout the work. Observe that most proteins show some direct or indirect interaction, except the transcription factors SOX11, ASCL1, and members of the KLF family (KLF1, 7, 12, 13, 14, 15, 16, and 19). Edges represent protein-protein associations, as follows: Known interactions—Light blue (from curated databases) and purple (experimentally determined); Predicted interactions—dark green (gene neighborhood), red (gene fusions), and dark blue (gene co-occurrence); Others—light green (text-mining), black (co-expression), and cyan (protein homology). Observe that most proteins coded by genes manipulated in previous work show some degree of association. The total number of edges is 104, whereas the expected number of edges for a random set of proteins of similar size is 24, indicating that the proteins are at least partially biologically connected, as a group.

Genetic manipulations of single targets genes promote some degree of axon regrowth in different types of neurons (Table 1; Figure 1). Still, functional recovery has been limited, indicating that combinatorial approaches may be necessary to induce robust axon regeneration, synapse formation and myelination, all required for efficient propagation of neuronal signals and functional recovery. Interestingly, most genes manipulated in experimental protocols aiming at boosting axonal regeneration show some degree of interaction (Figure 2) and are involved in the positive regulation of macromolecules biosynthesis (GO: 0010557). The transcription factors ASCL1 and SOX11 are exceptions to this rule, suggesting that these proteins may elicit axonal regeneration through a distinct mechanism.

The crosstalk among proteins involved in axon regeneration may help to explain the additional beneficial effects of combinatorial genetic manipulations on axon regeneration and neuronal survival. Indeed, co-deletion of PTEN and SOCS3, has produced one of the most robust effects on axon regrowth to date. While single deletion of either PTEN or SOCS3 induces axon regeneration of the optic nerve up to 2–3 mm distal to the lesion, co-deletion of PTEN and SOCS3 results in more than ten-fold increase in the number of regenerating axons at 2 mm distal to the lesion compared with deletion of either gene alone (Sun et al., 2011). Moreover, in the double mutants, a substantial number of regenerating axons reached the optic chiasm and several could grow even further, reaching the optic-tract brain entry zone, the hypothalamus and the suprachiasmatic nuclei area. In addition to axon regeneration, co-deletion of PTEN and SOCS3 also significantly enhances RGC survival compared to single deletion (Sun et al., 2011). In the spinal cord, deletion of SOCS3 promotes sprouting of uninjured CST axons after unilateral pyramidotomy, an effect that can be further enhanced by co-deletion of PTEN, resulting in significant recovery of skilled locomotion (Jin et al., 2015). These findings suggest that PTEN/mTOR and SOCS3/JAK/STAT3 pathways act synergistically to promote axon regeneration, sprouting and neuronal survival and point to these pathways as potential targets for combined manipulation in order to promote functional recovery. However, as mentioned before, both pathways are involved in tumorigenesis, thus long-term studies are necessary to prove the safety of PTEN and SOCS3 co-deletion.

Combinatorial modulation of SOCS3 and KLF family member of transcription factors have also promoted additional effects on axon regeneration. Qin and colleagues showed that conditional deletion of SOCS3 promotes axon regeneration after optic nerve lesion, which is further enhanced by co-deletion of KLF4. While in this study the authors observed beneficial effects of double-deletion on axon regeneration, RGC survival was not improved (Qin et al., 2013). However, in KLF4-deleted animals, intravitreal injection of CNTF, a well know cytokine that activates the JAK/STAT3 pathway, dramatic enhances axon regrowth and, interestingly, results in a significant increase in the survival of injured RGCs. Moreover, the authors showed that KLF4 physically interacts with STAT3 and suppresses STAT3-dependent gene expression (Qin et al., 2013). Therefore, these findings indicate a crosstalk between KLF4 and STAT3 that could be manipulated in a synergistic fashion to promote axon regrowth and neuronal survival.

In the same direction, Belin and colleagues observed that simultaneous overexpression of c-Myc and deletion of PTEN further increase axon regrowth and RGC survival compared with either strategy alone. The combination of PTEN and SOCS3 deletion with overexpression of CNTF and c-Myc promotes robust regeneration of injured optic nerve axons, with a five-fold increase in the number of regenerating axons at the proximal end of the optic chiasm, compared with co-deletion of PTEN and SOCS3 and overexpression of CNTF only (Belin et al., 2015). Despite the impressive regenerating effect obtained by this combinatorial approach, one important issue raised in this study was that regenerating axons also projected ectopically into the contralateral optic nerve, suggesting possible guidance problems. Using the visual system, Pernet and colleagues have shown that ROCK is an important protein involved in the control of axonal growth direction. Overexpression of STAT3 in RGC promotes axonal regeneration in the injured optic nerve. However, analysis of whole-mounted optic nerves in three dimensions showed that the regenerating axons displayed irregular courses, suggesting axonal misguidance. Pharmacological inhibition of ROCK reduced the misguidance issues of regenerating axons and improved long-distance axon regeneration of RGCs overexpressing STAT3 (Pernet et al., 2013), suggesting that correcting direction problems is an important step toward robust axon regeneration and pointing to ROCK as a pivotal target. It will be interesting to test in the future whether the manipulation of ROCK could improve the directional issues observed in the study by Belin and co-workers. Nevertheless, these work manipulated proteins involved in tumorigenesis, namely PTEN, SOCS3, and c-Myc, which again raises important concerns regarding the potential long-term effects of this manipulation.

Recently, combination of neuronal electrical stimulation and expression of proteins that promote axon regrowth has emerged as an alternative to boost regeneration. Enhancement of RGCs electrical activity using chemogenetic tools promotes axon regeneration after optic nerve lesion (Lim et al., 2016). Combination of increased RGC activity, by high-contrast visual stimulation, with genetic activation of the mTOR pathway promotes extensive axon regeneration (Lim et al., 2016). Moreover, triple combination of high-contrast visual stimulation, mTOR activation, and removal of the visual input from the intact eye (i.e., forcing the use of lesioned eye) leads to the regrowth of RGC axons along the entire optic pathway (Lim et al., 2016). In this combinatorial strategy, regenerating axons avoided incorrect targets and projected to their normal target nuclei partially rescuing visual behavior (Lim et al., 2016). These impressive findings show the importance of mTOR activation and, more interesting, the importance of neuronal activity to promote long distance axon regeneration and reconnection with the correct targets. Nonetheless, the number of axons that find their correct target is insufficient to promote complete recovery, indicating that additional advances are still necessary.

In addition to long-distance regeneration and reconnection to proper targets, re-myelination is also an import step to promote functional recovery. Manipulation of PTEN and SOCS3 expression robustly promotes axon regrowth and formation of functional synapses in the superior colliculus, but fails to produce significant recovery of visual function. This failure can be partly explained by the lack of myelination in regenerated axons, which exhibit poor electrical conductance. Accordingly, application of voltage-gated potassium channel blockers restores conduction and promotes recovery of behavioral functions (Bei et al., 2016). Thus, proper myelination of regenerating axons is a crucial step to improve nerve conduction to ultimately achieve functional recovery after injuries to the CNS.

## Perspectives

In the last decade, many efforts have been made to uncover the signaling pathways and networks involved in axon growth in the injured CNS. The use of different strategies to modulate gene expression, such as transgenic animals and viral vectors, provided useful tools to analyze the role of specific proteins in a variety of events during neuronal regeneration. Moreover, the possibility of temporal control of gene expression, using the CreERT/Tamoxifen system, can allow the evaluation of the role of specific molecules at different time-points after CNS injury. Finally, the use of AAV-mediated transduction to manipulate target genes in adult neurons after CNS injury and stimulate axon regeneration represents a fundamental step toward the translation of these techniques into clinic. Because of all advantages, including the ability to efficiently target various cell types in the CNS, the excellent safety profile and low immunogenicity that allows for long-term expression of the transgene after a single administration, recombinant AAV vectors are the most suited vector for gene manipulation in the CNS, which include the delivery of therapeutic genes or the Cre into transgenic animals to either overexpress or delete specific genes. Moreover, we believe that AAV are currently the most favored vectors for gene therapy in the injured CNS.

Optic nerve lesion and spinal cord injury are the most used experimental models employed to investigate the role of specific genes in axon regeneration in the CNS. The optic nerve lesion has many experimental advantages that facilitate the use of genetic tools and evaluation of the effects. On the other hand, spinal cord injury is more important clinically. Although, both models have advantages and disadvantages, both are very useful models to employ genetic techniques and identify genes involved in the control of axon regrowth.

Yet, although several groups identified many potential candidates to promote axon regrowth, our current understanding of the biological processes driving axon regeneration is still relatively limited, demonstrating that additional studies are necessary for a more accurate view of the mechanisms and synergistic effects of molecules controlling axon growth. Up to date, most molecular pathways tested in the adult CNS are involved in general processes of macromolecule biosynthesis and cell differentiation (Table 2). However, these processes are not specific for neurons and are often associated with cancer (Table 2). Thus, the identification of molecular mechanisms specific for axon growth, if they may exist, will likely contribute to the design of more efficient and safer strategies to promote CNS repair.

**Table 2:** First 20 functional enrichments in the network shown in Figure 2 using Biological Process Gene Ontology (GO) and Kyoto Encyclopedia of Genes and Genomes (KEGG) Pathways.

**#Pathway ID**	**Pathway description**	**Observed gene count**	**False discovery rate**	**Matching proteins in your network (labels)**
**BIOLOGICAL PROCESS GENE ONTOLOGY (GO)**				
GO.0010557	Positive regulation of macromolecule biosynthetic process	20	9.39e-13	Akt1,Ascl1,Atf3,Bdnf,Fos,Klf1,Klf12,Klf13,Klf14,Klf15,Klf4,Klf5,Klf7,Klf9,Mtor, Myc,Pten,Rhoa,Sox11,Stat1
GO.0010628	Positive regulation of gene expression	20	9.39e-13	Akt1,Ascl1,Atf3,Bdnf,Fos,Klf1,Klf12,Klf13,Klf14,Klf15,Klf4,Klf5,Klf7,Klf9,Mtor,Myc,Pten,Rhoa,Sox11,Stat1
GO.0045893	Positive regulation of transcription, DNA-templated	19	9.39e-13	Akt1,Ascl1,Atf3,Bdnf,Fos,Klf1,Klf12,Klf13,Klf14,Klf15,Klf4,Klf5,Klf7,Klf9,Mtor,Myc,Pten,Sox11,Stat1
GO.0031328	Positive regulation of cellular biosynthetic process	20	9.56e-13	Akt1,Ascl1,Atf3,Bdnf,Fos,Klf1,Klf12,Klf13,Klf14,Klf15,Klf4,Klf5,Klf7,Klf9,Mtor,Myc,Pten,Rhoa,Sox11,Stat1
GO.0045595	Regulation of cell differentiation	19	9.56e-13	Akt1,Ascl1,Bdnf,Cntf,Creb1,Fos,Klf13,Klf4,Klf5,Mtor,Myc,Pten,Rhoa,Rock1,Rock2,Socs2,Socs3,Sox11,Stat1
GO.0051173	Positive regulation of nitrogen compound metabolic process	20	9.56e-13	Akt1,Ascl1,Atf3,Bdnf,Fos,Klf1,Klf12,Klf13,Klf14,Klf15,Klf4,Klf5,Klf7,Klf9,Mtor,Myc,Pten,Rhoa,Sox11,Stat1
GO.0006355	Regulation of transcription, DNA-templated	23	2.09e-12	Akt1,Ascl1,Atf3,Bdnf,Fos,Klf1,Klf12,Klf13,Klf14,Klf15,Klf16,Klf4,Klf5,Klf6,Klf7,Klf9,Mtor,Myc,Pten,Rhoa,Sox11,Stat1,Stat2
GO.0031325	Positive regulation of cellular metabolic process	22	8.17e-12	Akt1,Ascl1,Atf3,Bdnf,Cntf,Fos,Klf1,Klf12,Klf13,Klf14,Klf15,Klf4,Klf5,Klf7,Klf9,Mtor,Myc,Pten,Rhoa,Sox11,Stat1,Stat2
GO.0007166	Cell surface receptor signaling pathway	18	9.16e-12	Akt1,Ascl1,Bdnf,Cntf,Cntfr,Creb1,Fos,Jun,Klf4,Klf6,Myc,Rhoa,Rock2,Socs1,Socs2,Socs3,Stat1,Stat2
GO.0006351	Transcription, DNA-templated	20	1.03e-11	Ascl1,Atf3,Creb1,Fos,Jun,Klf1,Klf12,Klf13,Klf14,Klf15,Klf16,Klf4,Klf5,Klf6,Klf7,Klf9,Myc,Sox11,Stat1,Stat2
GO.0010243	Response to organonitrogen compound	14	1.03e-11	Akt1,Ascl1,Creb1,Fos,Jun,Klf15,Klf4,Mtor,Myc,Pten,Rhoa,Socs2,Socs3,Stat1
GO.0018130	Heterocycle biosynthetic process	21	1.03e-11	Ascl1,Atf3,Creb1,Fos,Jun,Klf1,Klf12,Klf13,Klf14,Klf15,Klf16,Klf4,Klf5,Klf6,Klf7,Klf9,Mtor,Myc,Sox11,Stat1,Stat2
GO.0044271	Cellular nitrogen compound biosynthetic process	22	1.03e-11	Akt1,Ascl1,Atf3,Creb1,Fos,Jun,Klf1,Klf12,Klf13,Klf14,Klf15,Klf16,Klf4,Klf5,Klf6,Klf7,Klf9,Mtor,Myc,Sox11,Stat1,Stat2
GO.0048519	Negative regulation of biological process	25	1.03e-11	Ascl1,Atf3,Bdnf,Cntf,Cntfr,Creb1,Jun,Klf12,Klf13,Klf14,Klf15,Klf16,Klf4,Klf5,Klf9,Mtor,Myc,Pten,Rhoa,Rock1,Rock2,Socs1,Socs2,Socs3,Stat1
GO.0048522	Positive regulation of cellular process	25	1.96e-11	Ascl1,Atf3,Bdnf,Cntf,Cntfr,Fos,Klf1,Klf12,Klf13,Klf14,Klf15,Klf4,Klf5,Klf7,Klf9,Myc,Pten,Rhoa,Rock1,Rock2,Socs2,Socs3,Sox11,Stat1,Stat2
GO.0048523	Negative regulation of cellular process	24	2.03e-11	Ascl1,Atf3,Bdnf,Cntf,Cntfr,Creb1,Jun,Klf12,Klf13,Klf14,Klf15,Klf16,Klf4,Klf5,Klf9,Mtor,Myc,Pten,Rhoa,Rock1,Socs1,Socs2,Socs3,Stat1
GO.1901362	Organic cyclic compound biosynthetic process	21	2.04e-11	Ascl1,Atf3,Creb1,Fos,Jun,Klf1,Klf12,Klf13,Klf14,Klf15,Klf16,Klf4,Klf5,Klf6,Klf7,Klf9,Mtor,Myc,Sox11,Stat1,Stat2
GO.2000026	Regulation of multicellular organismal development	18	2.09e-11	Akt1,Ascl1,Bdnf,Cntf,Creb1,Fos,Jun,Klf13,Klf4,Mtor,Myc,Pten,Rhoa,Rock1,Rock2,Socs2,Sox11,Stat1
GO.0010604	Positive regulation of macromolecule metabolic process	21	2.21e-11	Akt1,Ascl1,Atf3,Bdnf,Cntf,Fos,Klf1,Klf12,Klf13,Klf14,Klf15,Klf4,Klf5,Klf7,Klf9,Mtor,Myc,Pten,Rhoa,Sox11,Stat1
GO.0030154	Cell differentiation	22	1.23e-10	Akt1,Atf3,Bdnf,Cntf,Creb1,Fos,Jun,Klf1,Klf15,Klf4,Klf5,Klf7,Mtor,Myc,Pten,Rhoa,Rock1,Rock2,Socs1,Socs2,Socs3,Stat1
**KYOTO ENCYCLOPEDIA OF GENES AND GENOMES (KEGG) PATHWAYS**				
4380	Osteoclast differentiation	8	1.5e-09	Akt1,Creb1,Fos,Jun,Socs1,Socs3,Stat1,Stat2
4630	JAK-STAT signaling pathway	8	2.65e-09	Akt1,Cntf,Cntfr,Myc,Socs1,Socs3,Stat1,Stat2
5161	Hepatitis B	8	2.65e-09	Akt1,Creb1,Fos,Jun,Myc,Pten,Stat1,Stat2
5200	Pathways in cancer	8	7.84e-07	Akt1,Fos,Jun,Mtor,Myc,Pten,Rhoa,Stat1
5210	Colorectal cancer	5	1.52e-06	Akt1,Fos,Jun,Myc,Rhoa
5206	MicroRNAs in cancer	6	1.79e-06	Mtor,Myc,Pten,Rhoa,Rock1,Socs1
4917	Prolactin signaling pathway	5	2.47e-06	Akt1,Fos,Socs1,Socs3,Stat1
4062	Chemokine signaling pathway	6	5.66e-06	Akt1,Rhoa,Rock1,Rock2,Stat1,Stat2
4510	Focal adhesion	6	1.15e-05	Akt1,Jun,Pten,Rhoa,Rock1,Rock2
4668	TNF signaling pathway	5	1.15e-05	Akt1,Creb1,Fos,Jun,Socs3
5205	Proteoglycans in cancer	6	1.44e-05	Akt1,Mtor,Myc,Rhoa,Rock1,Rock2
5166	HTLV-I infection	6	4.01e-05	Akt1,Atf3,Creb1,Fos,Jun,Myc
4921	Oxytocin signaling pathway	5	5.14e-05	Fos,Jun,Rhoa,Rock1,Rock2
4022	cGMP-PKG signaling pathway	5	6.83e-05	Akt1,Creb1,Rhoa,Rock1,Rock2
5164	Influenza A	5	6.83e-05	Akt1,Jun,Socs3,Stat1,Stat2
5132	Salmonella infection	4	7.69e-05	Fos,Jun,Rock1,Rock2
4012	ErbB signaling pathway	4	9.74e-05	Akt1,Jun,Mtor,Myc
5215	Prostate cancer	4	0.000111	Akt1,Creb1,Mtor,Pten
5168	Herpes simplex infection	5	0.000114	Fos,Jun,Socs3,Stat1,Stat2
4915	Estrogen signaling pathway	4	0.00013	Akt1,Creb1,Fos,Jun

We believe that a more comprehensive knowledge about the molecular control of axon elongation, axon guidance, synaptic formation, and myelination in the developing CNS may further contribute to this goal. As discussed in previous sections, the developmental decline in the capacity for axon growth in the CNS is associated with numerous changes in gene expression, pointing to the developing CNS as an interesting model to identify new targets involved in axon growth, in particular molecules controlling gene expression programs, i.e., transcription factors. Genetic manipulation of transcription factors that act as master key regulators of axon elongation during development might represent a valuable strategy for promoting axon regeneration in adults.

In addition to the identification of new targets, modifications of previous strategies to avoid undesired side effects should also be considered. For example, genetic tools designed to temporally control the expression of selected transcription factors, such as SOX11, may allow the stimulation of axon growth and by-pass the undesired effect of cell death. Similar approaches may be considered to the combination of PTEN, SOCS3, and c-Myc, which leads to a robust axon growth but are also associated with tumor formation. Therefore, long-term studies employing temporal control of gene expression are necessary to establish the efficiency and safety of such strategies to promote axon regeneration.

Last but not least, blocking axonal degeneration improves the ability of axons to regenerate past a lesion site (Ribas et al., 2017), demonstrating that axonal stabilization could be an interesting strategy to facilitate axon regeneration. Moreover, in addition to promote robust axon regeneration, other events, such as axon guidance to correct targets, establishment of functional synapses, and remyelination, are also important to successfully restore neuronal function. This is still a long road that will require many additional advances in our understanding of how different intrinsic and extrinsic signals interact to generate a precisely wired CNS. In sum, we advocate that the identification of key molecular players for axon growth and guidance during development, as well as a systematic analysis of their effects in the adult CNS after injury, will contribute to the design of successful therapeutic interventions aiming at the repair of the injured CNS in conditions of neurodegenerative diseases or physical trauma.

## Author contributions

VR and MC designed, wrote, revised, and finalized the manuscript. All authors read and approved the final version of the manuscript.

### Conflict of interest statement

The authors declare that the research was conducted in the absence of any commercial or financial relationships that could be construed as a potential conflict of interest.
